# Association of A561C and G98T Polymorphisms in E-Selectin Gene with Coronary Artery Disease: A Meta-Analysis

**DOI:** 10.1371/journal.pone.0079301

**Published:** 2013-11-18

**Authors:** Xiaoyan Wang, Junxiao Zhang, Xunbo Du, Minmin Song, Chongqi Jia, Huanliang Liu

**Affiliations:** 1 The Sixth Affiliated Hospital and Guangdong Institute of Gastroenterology, Sun Yat–sen University, Guangzhou, China; 2 Institute of Human Virology, Sun Yat–sen University, Guangzhou, China; 3 Key Laboratory of Tropical Disease Control (Ministry of Education), Sun Yat–sen University, Guangzhou, China; 4 Department of Human Genetics, Radboud University Nijmegen Medical Centre, Nijmegen, The Netherlands; 5 Chengdu Municipal Center for Disease Control and Prevention, Chengdu, China; 6 Department of Epidemiology and Health Statistics, Shandong University, Jinan, China; University of Oxford, United Kingdom

## Abstract

**Objective:**

E-selectin (SELE) mediates the rolling and adhesion of leukocytes on activated endothelial cells and plays a critial role in the pathogenesis of coronary artery disease (CAD). Associatons between the A561C and G98T polymorphisms of the *SELE* gene and CAD risk were investigated broadly, but the results were inconsistent. In the present study, we performed a meta-analysis to systematically evaluate the associations between the two polymorphisms and the risk of CAD.

**Methods:**

Comprehensive research was conducted to identify relevant studies. The fixed or random effect model was selected based on the heterogeneity among studies, which was evaluated with Q-test and *Ι^2^*. Meta-regression was used to explore the potential sources of between-study heterogeneity. Peters's linear regression test was used to estimate the publication bias.

**Results:**

Overall, 24 articles involving 3694 cases and 3469 controls were included. After excluding articles deviating from Hardy–Weinberg equilibrium in controls and sensitive analysis, our meta-analysis showed a significant association between the A561C ploymprphism and CAD in dominant (OR  = 1.84, 95% CI  = 1.56–2.16) and codominant (OR  = 1.74, 95% CI  = 1.49–2.03) models. As for the G98T polymorphism, significantly increased CAD risk was observed in dominant (OR  = 1.47, 95% CI  = 1.16–1.87) and codominant (OR  = 1.48, 95% CI  = 1.18–1.86) models, but after subgroup analysis, the association was not significant among Caucasians in dominant (OR  = 1.58, 95% CI  = 0.73–3.41) and codominant (OR  = 1.58, 95% CI  = 0.79–3.20) models.

**Conclusions:**

Despite some limitations, our meta-analysis suggested that the *SELE* gene polymorphisms (A561C, G98T) were significantly associated with increased risk of CAD. However, after subgroup analysis no significant association was found among Caucasians for the G98T polymorphism, which may be due to the small sample size and other confounding factors. Future investigations with multicenter, large-scale, and multi-ethnic groups are needed.

## Introduction

Coronary artery disease (CAD) has become a major cause of death and disability, accounting for up to 40% of all lethal events [1], and it is expected to be the leading cause of disease burden worldwide by 2020 [2]. CAD is a multifactorial disease resulting from environmental and genetic influences and their interactions [3], and genetic determinants contributing to huge amount of susceptibility to CAD. Strong evidence shows that in CAD, men with 2 or more affected first degree relatives (parents or siblings) have a 3.4 times increased risk of developing myocardial infarction [4], implying an important role of genetics. In recent years, a considerable number of candidate loci and genes for CAD have been revealed [5]–[8], however, progress in unraveling the genetic risk factors of CAD is still not fully understood and is the subject of intense investigation.

Inflammation plays a major role in pathogenesis of atherosclerosis [9], which is the pathogenesis of CAD. Development and progression of atherosclerosis involves recruitment and binding of circulating leukocytes to areas of inflammation within the vascular endothelium [10]. This progress is predominantly modulated and regulated by a diverse array of adhesion molecules including selectins, intergins, immunoglobins and chemokines [11], [12]. The family of selectins comprises E-, P- and L-selectins, which mediate the process of rolling and diapedesis, and are also involved in the promotion of atherosclerosis [13]. Experiments using E- and P-selectin double knockout mice suggest that E-and P-selectin together play an important role in both early and advanced stages of atherosclerotic lesion development [14].

E-selectin (SELE, endothelial leukocyte adhesion molecule, ELAM1, CD62E), is a surface glycoprotein molecule expressed on endothelial cells upon activation by cytokines [15]. SELE supports the rolling of leukocytes on activated endothelial cells and efficiently mediates the adhesion of circulating monocytes and lymphocytes to endothelial cells [16]. An increased expression of SELE has been observed in the arterial endothelium interacting with lymphocytes and macrophages in human atherosclerotic lesions [17]. The gene encoding SELE is located in chromosome 1q22-q25 (http://www.ncbi.nlm.nih.gov/gene/6401), consisting of 14 exons and 13 introns spanning about 13 kilobases of DNA. Several polymorphisms of the *SELE* gene have been mentioned, the following two polymorphisms were considered the most interesting, (1) the A561C polymorphism (rs5361) [18], a single base A to C transition polymorphism in exon 4, which results in an amino acid substitution serine to arginine at position 128 of EGF-domain of the SELE protein (Ser128Arg), has been known to increase the ligand-binding function of the protein [19], and (2) the G98T polymorphism (rs1805193), a G toT mutation in the untranslated region of SELE [20], may influence the expression of SELE [21].

Previously it was suggested that the *SELE* gene A561C and G98T polymorphisms might be associated with a predisposition to severe coronary or peripheral atherosclerosis (more than 50% stenosis of at least one major coronary or peripheral vessel because of atberosclerosis) [18], [20], and the correlations between the two polymorphisms and CAD have been reported in several ethnic groups, including Japanese [22], Arabs [23], Egyptians [24]and so on. However the above studies were mostly from single-centers with a small sample size and low reliability, and the results were often not reproducible and remain unknown. To elucidate the relationship between the two polymorphisms and its effect on CAD risk, we herein conducted a meta-analysis to (1) assess the effect of the *SELE* gene polymorphisms (A561C and G98T) on the risk of CAD; (2) evaluate the potential heterogeneity among studies; and (3) explore the potential publication bias.

## Materials and Methods

### Literature search strategy

A computer-based online retrieval was performed using the databases of PubMed, Web of Knowledge (ISI), China National Knowledge Infrastructure (CNKI), China Biology Medical literature database (CBM), Database of Chinese Scientific and Technical Periodicals (VIP) and Google Scholar covering the period from 1994 to August 2012. The following Medical Subject Headings (MeSH) were used as the search terms: “Cardiovascular Diseases”, “E-selectin”, and “Genetic Variation”. The folllowing keywords were also used in the search: “coronary artery disease”, “coronary heart disease”, “myocardial infarction”, “angina pectoris”, “ischemic heart disease”, “coronary death” and “E-selectin”, “SELE”, “CD62E”, “ELAM1” and “polymorphism”, “mut*”, “varia*”. We further checked the reference lists of the relevant papers we identified, including reviews and meta-analysis, to discover other relevant studies that were not captured initially, and contacted the authors of published papers directly, if crucial data was not reported in the original papers. We restricted search results to papers published in English or Chinese.

### Selection criteria

Studies included in our meta-analysis had to be in accordance with the following criteria: (1) case-control or cohort study published in an original study aimed to explore the associations of the A561C and/or G98T in the *SELE* gene and susceptibility to CAD; (2) sufficient genotype data in case and control groups in a case–control study or exposed and unexposed groups in a cohort study was provided or can be calculated; (3) subjects of each study group should come from the same time period and ethnicity; (4) If there were multiple publications from the same study group, the most recent or complete study with the largest sample size was used; (5) The diagnosis of CAD was definite, and relevant outcomes in cases should be confirmed based on WHO criteria or coronary arteriography (minimally 50% stenosis of at least one major coronary artery).

All studies were reviewed by two investigators independently to identify the eligible studies included in this meta-analysis. In case of disagreement, consensus was obtained with a third reviewer by joint review of the study.

### Data extraction

Study eligibility was determined based on the selection criteria, and data was entered into a preformatted spreadsheet by two independent investigators who reached a consensus on all of the items. Data extracted from eligible studies were as follows: the first author, year of publication, country, ethnic origin, numbers of case (exposed) and control (unexposed) groups, genotype and allele distributions, the variant allele frequency in control (unexposed) groups, mean age, male percentage in case (exposed) and control (unexposed) groups. For studies including subjects from different types of populations, data was extracted separately from each population.

### Quantitative data synthesis

Chi-squared analysis with exact probability was used to assess deviation from the Hardy-Weinberg equilibrium (HWE) for the A561C and G98T genotype distributions of the *SELE* gene in controls with the significance set at *P*<0.05. The inverse-variance weighted mean of the logarithm of Odds Ratio (OR) with 95% confidence intervals (CI) was calculated to assess the strength of the associations between the *SELE* gene A561C, G98T polymorphisms and risk of CAD. The pooled ORs were calculated by using the dominant model (CC + AC versus AA for the A561C polymorphism, TT + GT versus GG for the G98T polymorphism) and the codominant model (allele C versus allele A for the A561C polymorphism; allele T versus allele G for the G98T polymorphism). Between-study heterogeneity was calculated by the Chi-square-based *Q*-test and considered significant if *P*<0.05 [25]. *Ι^2^* of Higgins and Thompson [26] was also used to quantify the between-study heterogeneity. *Ι^2^* measures the percentage of variability in point estimates that is due to heterogeneity rather than sampling error or chance. If substantial heterogeneity (*P_heterogeneity_* <0.05) was observed among the studies, the DerSimonian and Laird random effect model (REM) was used to estimate the pooled OR. Otherwise, the Mantel–Haenszel fixed effect model (FEM) was adopted. Meta-regression with restricted maximum likelihood estimation [27] was used to identify the sources of heterogeneity, and the following potential covariates are included: ethnicity (categorised as Asian and Caucasian populations), publication year, sex (ratio of male percentage in case group to that in control group), and age (ratio of mean age in case group to that in control group). Subgroup analysis by ethnicity (categorised as Asian and Caucasian populations) was also carried out. Genetic variants causally associated with complex diseases will have small effects (risk ratios mostly <2.0) [3], [28], thereby we performed sensitive analysis by excluding the studies with OR >3.0, aiming to control the impact of outlier values on the pooled effect resulting from low cell counts within each single study [29]. Publication bias of the literature was assessed using modified Egger's linear regression test proposed by Peters et al. [30]. An influence analysis [31] was performed by omitting one study in each turn to assess the influence of an individual data set on the pooled ORs. If the point estimate of an individual study's omitted analysis lies outside the 95% CI of the pooled analysis, it is likely to have an excessive influence. All statistical analyses were performed with STATA 11.2 software (Stata Corporation, College Station, TX, USA). Two-tailed *P*<0.05 was accepted as statistically significant.

## Results

### Characteristics of the studies

**Figure 1:**
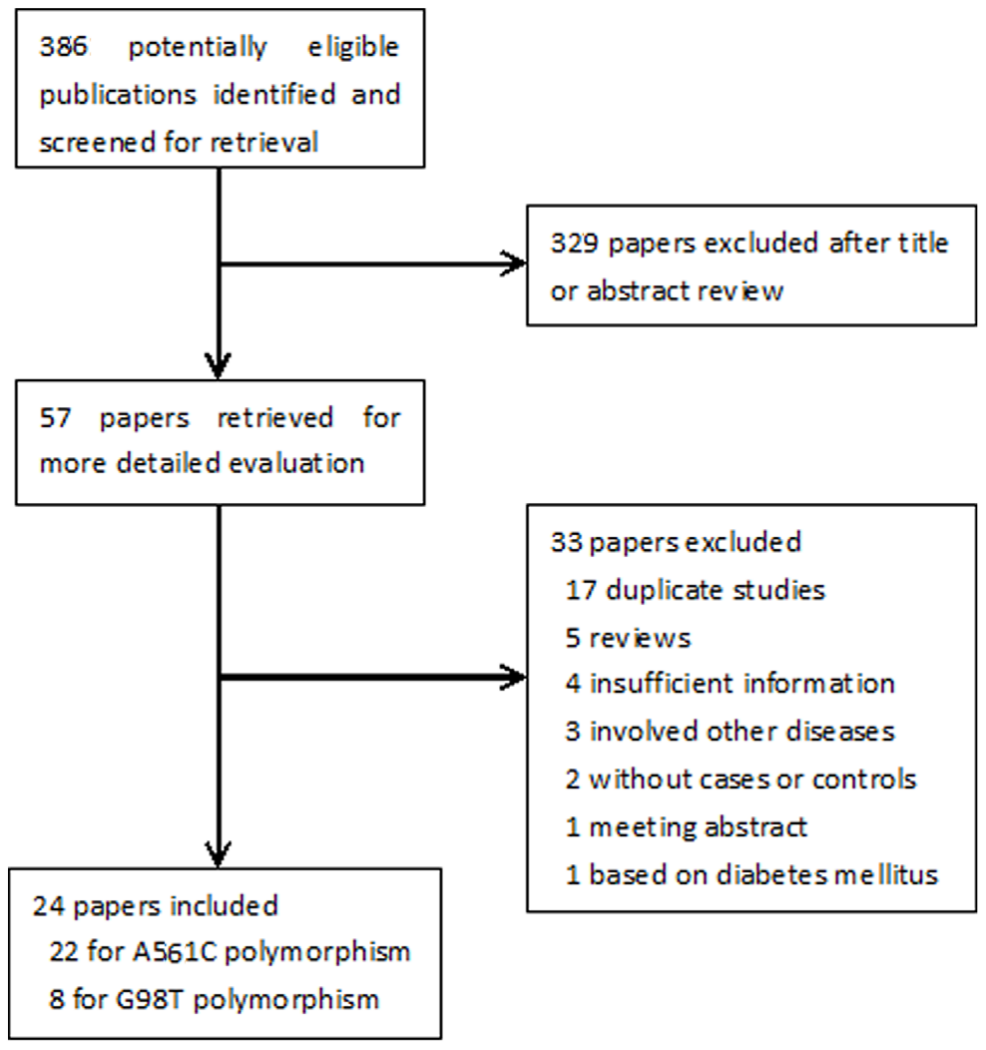
Flow diagram of search strategy and study selection.

The initial search yielded 386 potentially eligible papers based on the above search criteria, 329 papers were excluded after title or abstract review, and 33 additonal papers were excluded due to duplicate studies, reviews, studies with insufficient information and so on. Finally, 24 [22]–[24], [32]–[52] articles were included in the meta-analysis. A flow diagram schematizing the inclusion and exclusion process of identified articles with the inclusion criteria is presented in Fig 1.

The included papers were all case-control studies involving 3694 cases and 3469 controls. Among the 24 papers involving 32 outcomes, 21 papers involving 23 outcomes were for the A561C polymorphism and 8 papers involving 9 outcomes were for the G98T polymorphism. In most of the studies the polymorphisms in the controls were found to occur in frequencies consistent with HWE, with the exception of one article [44]. Articles [39], [45] including two groups with sufficient information were included as two independent outcomes. General characteristics and genotype distributions of the included studies are summarized in Table 1–2.

**Table 1:** Characteristics of the *SELE* gene A561C polymorphism genotype distributions in studies included in this meta-analysis.

First author	Year	Country	Ethnicity	Case	N	Genotypes AA/AC/CC		F	% of male		Mean age		*P* a
						Case	Control		Case	Control	Case	Control	
Wenzel [32]	1997	Germany	Caucasian	CHD	216	79/33/1	87/14/2	0.087	88	90	42^b^	38^b^	0.13
Ye [33]	1999	America	Caucasian	CAD	153	52/28/2	56/15/0	0.106	57	45	Na	Na	0.32
Lin [34]	2001	China	Asian	CAD	177	71/20/1	77/8/0	0.047	73	65	63.9	60.2	0.65
Yoshida [22]	2003	Japan	Asian	MI	462	118/17/0	305/22/0	0.034	76	70	57.7	47.6	0.53
Luo [37]	2003	China	Asian	CHD	190	74/17/2	87/10/0	0.052	87	89	54.0	56.0	0.59
Li [39] ^c^	2004	China	Asian	MI	388	170/28/0	177/13/0	0.034	76	76	66.1	67.8	0.63
Li [39] ^c^	2004	China	Asian	MI	372	162/25/0	173/12/0	0.032	79	80	56.7	54.7	0.65
Wei [40]	2004	China	Asian	AP	280	119/16/0	138/7/0	0.024	64	64	61.7	60.5	0.77
Huang [36]	2004	China	Asian	AP	315	132/23/0	149/11/0	0.034	72	72	61.7	60.7	0.65
Abu-Amero [23]	2006	Saudi Arabia	Caucasian	CAD	793	451/92/13	208/28/1	0.063	71	44	50.0	50.0	0.96
Hu [41]	2006	China	Asian	CHD	410	162/33/2	202/11/0	0.026	57	59	62.3	61.5	0.70
Jiang [42]	2006	China	Asian	CHD	289	89/52/4	130/14/0	0.049	66	61	61.7	59.7	0.54
Hamid [43]	2007	Egypt	Caucasian	CAD	50	23/5/2	18/2/0	0.05	80	80	45.7	47.8	0.81
Wei [44]	2007	China	Asian	AMI	368	147/20/1	182/16/2	0.05	61	60	64.0	63.0	0.03*
Ma [45] ^d^	2008	China	Asian	CHD	204	85/22/1	87/9/0	0.047	100	100	65.4	66.1	0.63
Ma [45] ^d^	2008	China	Asian	CHD	92	30/12/0	45/5/0	0.05	100	100	69.8	67.8	0.71
Zak [46]	2008	Poland	Caucasian	CAD	394	149/38/4	161/40/2	0.108	67	76	43.8	35.3	0.78
Zeng [47]	2008	China	Asian	CHD	410	196/23/1	182/8/0	0.021	62	62	65.0	63.0	0.77
Tripathi [49]	2009	India	Asian	CAD	660	273/56/0	294/37/0	0.056	79	74	57.1	53.2	0.28
Hong [48]	2009	China	Asian	CHD	82	53/13/0	14/2/0	0.063	Na	Na	Na	Na	0.79
Sakowicz [50]	2010	Poland	Caucasian	MI	298	116/41/5	102/29/5	0.143	83	47	40.9	54.3	0.12
Fang [51]	2011	China	Asian	ACS^e^	82	30/5/4	41/2/0	0.023	Na	Na	Na	Na	0.88
Motawi [24]	2012	Egypt	Caucasian	CAD	150	51/21/3	69/6/0	0.04	51	33	56.7	59.9	0.72

CHD: coronary heart disease, CAD: coronary artery disease, MI: myocardial infarction, AP: angina pectoris, AMI: acute myocardial infarction.

ACS: acute coronary syndrome, N: total number of subjects in each study, F: the C allele frequency in control, Na: not available. * *P*<0.05.

a *P* for Hardy–Weinberg equilibrium in the control group.

b Only the median age of cases and controls was available from the original article.

c/d One study with different populations.

e ACS mainly included unstable angina pectoris, myocardial infarction, coronary death in the original article.

**Table 2:** Characteristics of the *SELE* gene G98T polymorphism genotype distributions in studies included in this meta-analysis.

First author	Year	Country	Ethnicity	Case	N	Genotypes GG/GT/TT		F	% of male		Mean age		*P* a
						Case	Control		Case	Control	Case	Control	
Zheng [35]	2001	American	Caucasian	CAD	101	31/18/2	40/10/0	0.1	54.9	42	Na	Na	0.43
Luo [37]	2003	China	Asian	CHD	190	72/15/6	85/11/1	0.067	87.1	88.7	54.0	56.0	0.36
Li [38]	2004	China	Asian	CHD	437	210/27/1	181/18/0	0.045	71.0	70.4	63.5	61.9	0.50
Ma [45] b	2008	China	Asian	CHD	204	90/18/0	83/13/0	0.068	100.0	100.0	65.4	66.1	0.48
Ma [45] b	2008	China	Asian	CHD	92	35/7/0	41/9/0	0.09	100.0	100.0	69.8	67.8	0.48
Zak [46]	2008	Poland	Caucasian	CAD	394	150/37/4	164/37/2	0.101	66.5	75.9	43.8	35.3	0.96
Zeng [47]	2008	China	Asian	CHD	410	187/33/0	174/16/0	0.042	61.8	62.1	65.0	63.0	0.55
Hong [48]	2009	China	Asian	CHD	82	58/8/0	11/5/0	0.156	Na	Na	Na	Na	0.46
Zhang [52]	2011	China	Asian	CHD	328	150/26/0	141/11/0	0.036	61.9	66.7	62.9	63.7	0.64

CHD: coronary heart disease, CAD: coronary artery disease, N: total number of subjects in each study, F: the T allele frequency in control.

Na: not available.

a *P* for Hardy –Weinberg equilibrium in control group.

b One study with different populations.

### Association of the *SELE* gene A561C polymorphism with CAD risk

As shown in Table 3, we found that the C allele was significantly associated with increased CAD risk in dominant (REM: OR  = 2.23, 95% CI  = 1.83–2.71) and codominant (REM: OR  = 2.14, 95% CI  = 1.76–2.60) models. The association was stable when using the cumulative meta-analysis according to the publication year and sample size. In the subgroup analysis by ethnicity, for Caucasian populations, the C allele was found to be significantly associated with increased CAD risk in the dominant (REM: OR  = 1.78, 95% CI  = 1.25–2.54) and codominant (REM: OR  = 1.74, 95% CI  = 1.24–2.44) models. For the Asian ethnicity, the associaton was also found to be significant in the dominant (FEM: OR  = 2.49, 95% CI  = 2.06–3.00) and codominant (FEM: OR  = 2.40, 95% CI  = 2.01–2.87) models. After excluding one article [44] that deviated from HWE in controls, the associations were not altered appreciably.

**Table 3:** Pooled measures on the relationship of the *SELE* gene A561C polymorphism with CAD.

Data	Population	Model	Before Sensitive Analysis				After Sensitive Analysis			
			Pooled OR (95% CI)		*I* ^2^	*P*_h_	Pooled OR (95% CI)		*I* ^2^	*P*_h_
			FEM	REM	(%)		FEM	REM	(%)	
All includedarticles	Overall	Dominant	2.12 (1.83–2.44)**	2.23 (1.83–2.71)**	39.6	0.028	1.81 (1.55–2.12)**	1.80 (1.54–2.11)**	0	0.687
		Codominant	2.03 (1.78–2.33)**	2.14 (1.76–2.60)**	43.9	0.013	1.73 (1.49–2.00)**	1.71 (1.48–1.99)**	0	0.645
	Asian	Dominant	2.49 (2.06–3.00)**	2.48 (2.02–3.04)**	12.3	0.312	2.08 (1.69–2.57)**	2.07 (1.67–2.11)**	0	0.966
		Codominant	2.40 (2.01–2.87)**	2.38 (1.93–2.92)**	18.5	0.242	1.99 (1.62–2.43)**	1.97 (1.61–2.42)**	0	0.965
	Caucasian	Dominant	1.66 (1.32–2.08)**	1.78 (1.25–2.54)**	51.0	0.057	1.52 (1.20–1.93)**	1.53(1.17–2.00)**	15.5	0.314
		Codominant	1.62 (1.32–2.00)**	1.74 (1.24–2.44)**	55.0	0.038	1.47 (1.18–2.00)**	1.47 (1.14–1.90)**	24.0	0.262
Excluded forDHWE	Overall	Dominant	2.15 (1.86–2.50)**	2.28 (1.86–2.80)**	40.3	0.027	1.84 (1.56–2.16)**	1.83 (1.55–2.15)**	0	0.653
		Codominant	2.06 (1.80–2.37)**	2.19 (1.78–2.68)**	45.4	0.011	1.74 (1.49–2.03)**	1.73 (1.48–2.01)**	0	0.588
	Asian	Dominant	2.89 (2.14–3.16)**	2.57 (2.10–3.14)**	3.6	0.412	2.16 (1.73–2.70)**	2.15(1.72–2.69)**	0	0.984
		Codominant	2.50(2.07–3.01)**	2.47 (2.00–3.04)**	14.3	0.293	2.05 (1.65–2.54)**	2.04 (1.64–2.52)**	0	0.973
	Caucasian	Dominant	1.66 (1.32–2.08)**	1.78 (1.25–2.54)**	51.0	0.057	1.52 (1.20–1.93)**	1.53 (1.17–2.00)**	15.5	0.314
		Codominant	1.62 (1.32–2.00)**	1.74 (1.24–2.44)**	55.0	0.038	1.47 (1.18–1.82)**	1.47 (1.14–1.90)**	24.0	0.262

Dominant model: CC + AC versus AA; Codominant model: C versus A.

FEM: fixed effect model, REM: random effect model, DHWE: deviated from Hardy–Weinberg equilibrium.

** *P*<0.01.

*P*_h_: *P* value of *Q*-test for heterogeneity test.

### Association of the *SELE* gene G98T polymorphism with CAD risk

**Figure 2:**
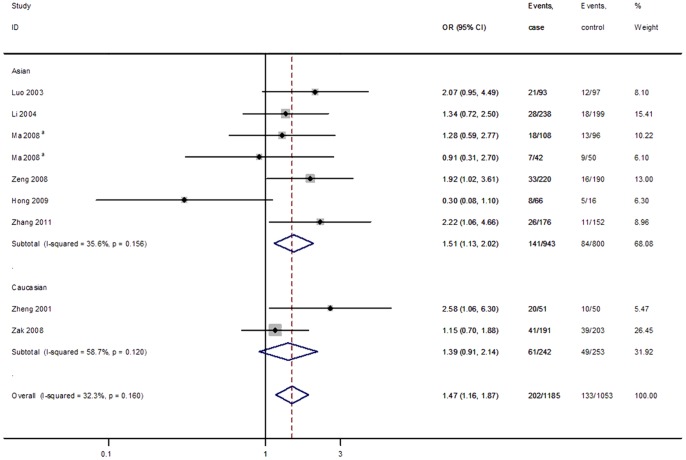
Forest plot of ORs for CAD in the dominant model (TT + GT vs.GG) of the *SELE* gene G98T polymorphism stratified by ethnicity. White diamonds denote the pooled ORs in the fixed effect. Black squares indicate the OR in each study, with square sizes inversely proportional to the standard error of the OR. Horizontal lines represent 95% CIs. ^a^One study with different types of populations.

As summarized in Table 4, the current meta-analysis showed that the T allele was significantly associated with increased CAD risk in the dominant (FEM: OR  = 1.47, 95% CI  = 1.16–1.87) and codominant (FEM: OR  = 1.48, 95% CI  = 1.18–1.86) models. However, subgroup analysis by ethnicity showed no significant association in dominant (REM: OR  = 1.58, 95% CI  = 0.73–3.41) nor codominant (REM: OR  = 1.58, 95% CI  = 0.79–3.20) models for Caucasian populations. All included articles of this meta-analysis were in HWE in controls. Fig 2 shows the forest plot of ORs in the dominant model (GT + TT vs GG) of the *SELE* gene G98T polymorphism.

**Table 4:** Pooled measures on the relationship of the *SELE* gene G98T polymorphism with CAD.

Population	Inherited Model	Pooled OR (95% CI)				*I^2^*	*P*_h_
		FEM	*P*	REM	*P*	(%)	
**Overall**	Dominant	1.47 (1.16–1.87)	0.002	1.46 (1.07–1.97)	0.016	32.3	0.160
	Codominant	1.48 (1.18–1.86)	0.001	1.46 (1.09–1.96)	0.011	33.3	0.151
**Asian**	Dominant	1.51 (1.13–2.02)	0.005	1.43 (0.98–2.08)	0.061	35.6	0.156
	Codominant	1.51 (1.14–2.00)	0.004	1.43 (0.99–2.07)	0.056	37.0	0.146
**Caucasian**	Dominant	1.39 (0.91–2.14)	0.126	1.58 (0.73–3.41)	0.249	58.7	0.120
	Codominant	1.43 (0.97–2.10)	0.073	1.58 (0.79–3.20)	0.199	58.9	0.119

Dominant model: TT + GT versus GG; Codominant model: T versus G.

FEM: fixed effect model, REM: random effect model.

*P*_h_: *P* value of *Q*-test for heterogeneity test.

### Sensitive analysis and sources of heterogeneity

**Figure 3:**
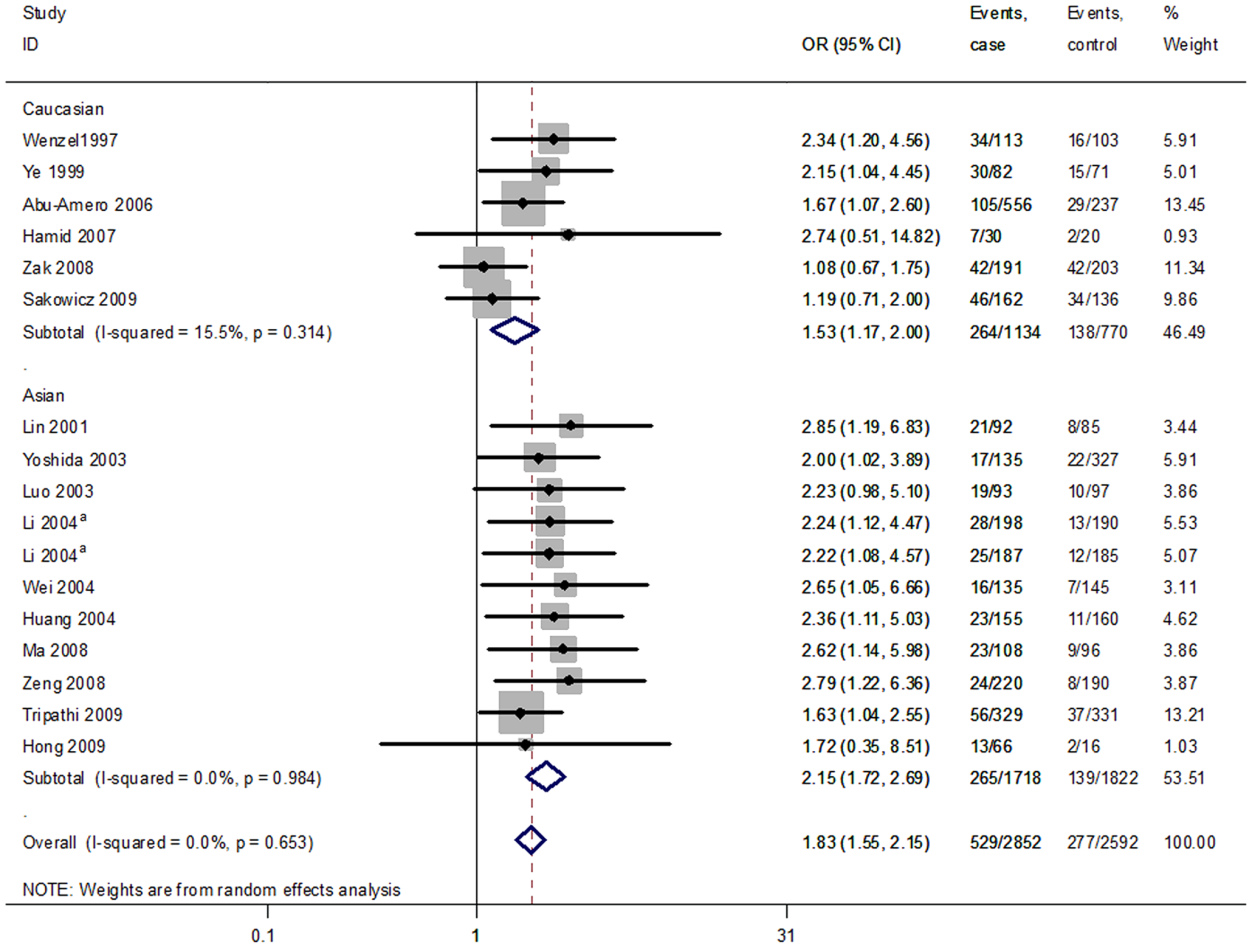
Forest plot of ORs for CAD in the dominant model (CC + AC vs.AA) of the *SELE* gene A561C polymorphism stratified by ethnicity. White diamonds denote the pooled ORs in the fixed effect. Black squares indicate the OR in each study, with square sizes inversely proportional to the standard error of the OR. Horizontal lines represent 95% CIs. ^a^One study with different types of populations.

For the A561C polymorphism, after exclusion of the article that deviated from HWE in controls and the sensitive analysis, the C allele associated with CAD remained significant in dominant (FEM: OR  = 1.84, 95CI  = 1.56–2.16) and codominant (FEM: OR  = 1.74, 95% CI  = 1.49–2.03) models. After the subgroup analysis, the associations were not altered substantially. Fig 3 shows the forest plot of ORs in the dominant model (AC + CC vs AA) of the *SELE* gene A561C polymorphism after excluding articles deviating from HWE in controls and the sensitive analysis.

Strong evidence of heterogeneity (*P_heterogeneity_* <0.05) among studies was demonstrated in dominant and codominant models considering the association of the A561C polymorphism with CAD after exclusion of articles deviating from HWE in controls. In order to systematically assess the heterogeneity among the studies, univariate meta-regression was conducted to explore the potential sources. Among the covariates of ethnicity, publication year, sex, and age for the A561C polymorphism, no covariates had a significant impact on the between-study heterogeneity with the *P* value of the above covariates being 0.05, 0.90, 0.41 and 0.78 separately in the dominant model. No significant between-study heterogeneity was found in the above-mentioned inherited models considering the association of the G98T polymorphism with CAD.

### Influence analysis

**Figure 4:**
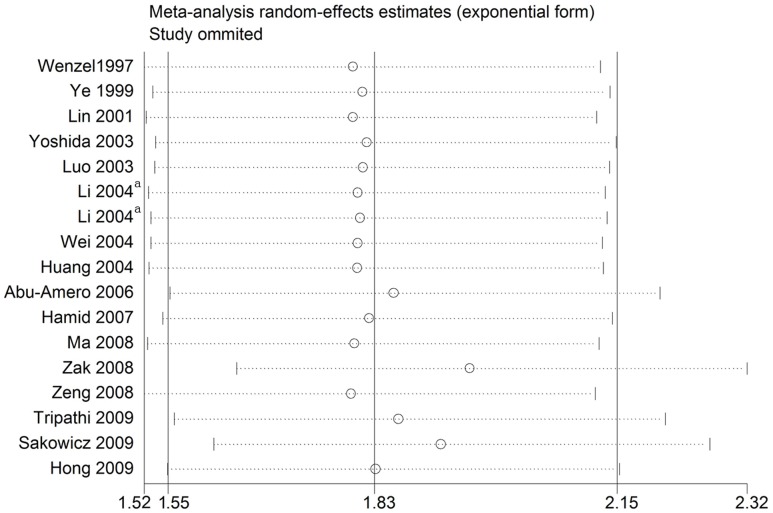
Analysis of the influence of individual studies on the pooled estimate in the dominant model (CC + AC vs. AA) of the *SELE* gene A561C polymorphism. Open circles indicate the pooled OR, given the named study is omitted. Horizontal lines represent the 95% CIs. ^a^One study with different types of populations.

**Figure 5:**
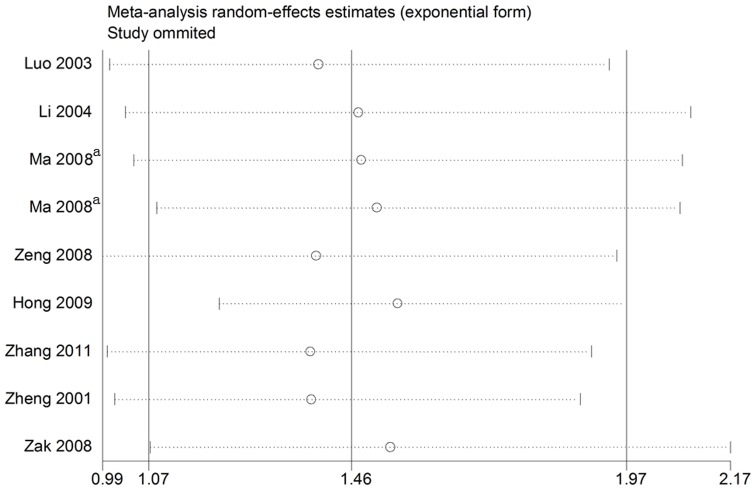
Analysis of the influence of individual studies on the pooled estimate in the dominant model (TT + GT vs. GG) of the *SELE* gene G98T polymorphism. Open circles indicate the pooled OR, given the named study is omitted. Horizontal lines represent the 95% CIs. ^a^One study with different types of populations.

As shown in Fig 4–5, after exclusion of articles deviating from HWE in controls and the sensitive analysis, no individual study was found to have excessive influence on the pooled effect in any of the dominant and codominant models considering the associations of the two aboved mentioned polymorphisms with CAD.

### Publication bias evaluation

Peters's linear regression test was performed to estimate the publication bias of the literature. For the A561C polymorphism, after exclusion of articles deviating from HWE in controls and the sensitive analysis, no statistically significant difference (*P* = 0.22 for CC + AC vs AA, *P* = 0.19 for C vs A allele) was found, with similar results for the G98T polymorphism (*P* = 0.33 for TT + GT vs GG, *P* = 0.54 for T vs G allele), indicating low publication bias in the current meta-analysis.

## Discussion

Atherosclerosis and CAD may be inflammatory conditions and SELE plays a key role in the initial process of inflammation. Observational studies have shown that elevated levels of SELE have been observed in acute myocardial infarction [53], coronary heart disease [54], restenosis following peripheral arterial angioplasty [55], and stable and unstable angina [56]. As we all know, polymorphisms of the genes encoding products involved in the atherosclerotic process play a crucial role in the etiology of atherosclerosis and CAD, thereby attention is being focused on the possibility that polymorphisms relate to the pathogenesis of atherosclerosis and cardiovascular disease. Recently, results from a meta-analysis reported that the *SELE* gene A561C polymorphism was significantly associated with susceptibility to ischemic stroke, which results from cerebrovascular atherosclerosis [57]. Several polymorphisms in the *SELE* gene, such as A561C [32], G98T [35], L554F [20], G2692A and C1091T [58], were successively reported to be associated with atherosclerosis and CAD. Sarecka-Hujar et al. suggested that the two SNPs of the *SELE* gene, A561C and G98T, were likely to control the SELE expression [21]. We herein come to a hypothesis that the two polymorphisms may be associated with coronary heart disease.

For the A561C polymorphism, some studies, such as Wenzel [32], Lin [34], Luo [37], Li [39], etc, showed an increased CAD risk with the C allele. Mechanistic studies have shown that this mutation exhibits dramatically decreased binding specificity while increasing affinity for additional ligands, resulting in a 2- to 3-fold increase in cellular adhesion. The C allele may increase leukocyte adherence to activated endothelium in areas susceptible to atherosclerotic plaque formation, therefore contributing to the progression of atherosclerosis and CAD [19]. Others, such as Abu-Amero [23], Sakowicz [50], ect, however, didn't find such an association between this polymorphism and CAD risk. Considering the different ethnicities, sample sizes, characteristics of the sample, which can introduce heterogeneity, it is difficult to identify the definite association between the A561C polymorphism and CAD, thus we conducted a meta-analysis to provide more credible evidence by systematically summarizing existing data.

In our meta-analysis, a total of 3518 cases and 3317 controls from 23 populations were included, however most of the populations were Asian (12 from China, 1 from Japan, 1 from India). A significant association was found between the C allele and CAD risk in the dominant and codominant models. After subgroup analysis by ethnicity, the assosiation was also significant in Asian and Caucasian populations. Strong evidence of heterogeneity (*P_heterogeneity_* <0.05) among studies was demonstrated in the dominant and codominant models, and we then performed an univariate meta-regression to explore the potential sources, including ethnicity, publication year, sex, and age, we found that no covariates above had a significant impact on the between-study heterogeneity. However, as we all know, CAD has a complex aetiology and pathophysiology generated by the combined effects of genes and environmental factors. Numerous genetic and environmental variables, as well as their interaction, may be potential contributors to this disease-effect unconformity. Besides an indeterminate number of characteristics that vary among studies, confounding factors could further contribute to between-study heterogeneity.

According to the SNP database at the NIH (http://www.ncbi.nlm.nih.gov/projects/SNP/snp_ref.cgi?rs=5361) and our present study, which shows that the distribution of the C allele frequency in controls was 0.04 and 0.09 in Asian and Caucasian populations separately, the C allele frequency in the Asian populations is significantly lower than that in the Caucasian populations. Considering that the different genetic background may contribute to the between-study heterogeneity, we performed subgroup analysis by ethinicity categorised as Asian and Caucasian, though in our present meta-analysis ethnicity was not found to be responsible for the between-study heterogeneity.

Another polymorphism of the *SELE* gene, the G98T mutation, was previously reported to be associated with a higher risk for early severe atherosclerosis in 99 cases and 100 controls in German Caucasians [20]. Recently Zeng [47] and Zhang [52] showed that G98T polymorphism was associated with CAD in Chinese, and the T allele may be a risk factor of CAD. However, the results of some articles, such as Luo [37], Li [38] etc, did not find such an association. In our meta-analysis of the G98T polymorphism, which included a total of 1185 cases and 1053 controls from 9 populations, a significant association with increased CAD risk was found in the dominant and codominant models. It is possible that the small sample size may not give adequate power to detect the slight effects of the mutation on CAD, therefore after subgroup analysis, no significant association was found in dominant and codominant models focusing on Caucasians, which merely included two articles involved in 242 cases and 253 controls. Other confounding factors between the two subgroups may also contribute to the pooled estimated effect. No significant between-study heterogeneity and publation bias were found in the above-mentioned inherited models considering the association of the G98T polymorphism with CAD.

Evidence has shown that comparing with an individual polymorphism that might not act independently to affect the susceptibility to a complex disease such as CAD, two or more neighboring SNPs or an interaction of the SNPs within haplotypes could be a major determinant of disease susceptibility [59], [60]. Thus, studies have revealed that the G98T and A561C mutations are in strong positive linkage disequilibrium in Caucasian populations [20], [46], [61] and there are synergistic effects between the two variants and hypercholesterolemia in determining CAD [46]. This suggests that the two variant alleles which could define the [C^561^-T^98^] haplotype or the interaction with other SNPs within other haplotypes might be associated with CAD. Moreover Wu [62] showed a significant association of a *SELE* haplotypes with the level of circulating E-selectin, which may be a functional variation. However Zheng [35] did not obeserve such a relationship between the two mutations in American populations and no evidence indicated the correlation between the two mutations in Asian populations. Thereby further studies estimating the effect of these two SNPs and other mutations in CAD along with gene-environment interactions may provide a better, comprehensive understanding of the associations. Besides according to the SNP database at the NIH, the C and T allele frequencies were different between Asian and Caucasian populations, as evidence revealed that the linkage disequilibrium and haplotype were both locus and population specific and could be affected by the local recombination rate, thus the defined haplotype in our study [C^561^-T^98^] could be different in different populations and these inter-ethnic differences in frequencies of SNPs and haplotypes may help to explain inconsistencies that have been reported in association studies [63], [64].

Though we included the latest data, several potential limitations must also be noticed in our meta-analysis. First, our search results were restricted to publications in Chinese and English, other relevant published and unpublished studies, which are likely to have null results, were not included. Second, most of the included studies were involved in Asian populations, primarily East Asian, especially when considering the association between the G98T mutation and CAD. Third, the lack of original data limited further evaluation of potential gene-gene and gene-environment interactions, which may be confounding factors to the pooled effect. Fourth, no covariates were found to be the significant sources of heterogeneity across studies on the A561C polymorphism and CAD risk, however, as we mentioned above, other possibilities, such as variations in design quality, genotyping method, and lifestyle factors, etc. may also contribute to between-study heterogeneity. Fifth, we did not find significant publication bias in our meta-analysis, which may be due to the small number of studies in the meta-analysis, especially for the G98T polymorphism which was only analyzed in 9 populations.

In conclusion, a significant association existed between the A561C polymorphism of the *SELE* gene and CAD risk with the AC/CC genotype and C allele conferring susceptibility for CAD, especially in Asian populations. Our meta-analysis also provided evidence that the G98T polymorphism was significantly associated with CAD risk, but was not significant in Caucasian populations, which may be due to the small sample size. However the confounding factors and biases still exist, and further studies are warranted to firmly validate the correlation between the *SELE* gene polymorphism and CAD through multicenter, large-scale, and multi-ethnic studies.

## Supporting Information

Checklist S1 **PRISMA Checklist.**
(DOC)
